# Assessment of stress responses in rhesus macaques (*Macaca mulatta*) to daily routine procedures in system neuroscience based on salivary cortisol concentrations

**DOI:** 10.1371/journal.pone.0190190

**Published:** 2018-01-02

**Authors:** Dana Pfefferle, Sina Plümer, Leonore Burchardt, Stefan Treue, Alexander Gail

**Affiliations:** 1 Welfare and Cognition Group, Cognitive Neuroscience Laboratory, German Primate Center–Leibniz Institute for Primate Research, Göttingen, Germany; 2 Leibniz-ScienceCampus Primate Cognition, German Primate Center & University of Göttingen, Germany; 3 Bernstein Center for Computational Neuroscience, Göttingen, Germany; CEA, FRANCE

## Abstract

Non-human primates participating in neurophysiological research are exposed to potentially stressful experimental procedures, such as dietary control protocols, surgical implants and their maintenance, or social separation during training and experimental session. Here, we investigated the effect of controlled access to fluid, surgical implants, implant-related cleaning of skin margins, and behavioral training sessions on salivary cortisol levels of adult male rhesus macaques participating in neurophysiological research. The animals were trained to chew flavored cotton swabs to non-invasively collect saliva samples. Our data show no differences in cortisol levels between animals with and without implants, but both, controlled access to fluid and cleaning of implants individually increased salivary cortisol concentrations, while both together did not further increase the concentration. Specifically, before cleaning, individuals with controlled access to fluid had 55% higher cortisol concentrations than individuals with free access to fluid. Under free access to fluid, cortisol concentrations were 27% higher after cleaning while no effect of cleaning was found for individuals under controlled fluid access. Training sessions under controlled access to fluid also did not affect salivary cortisol concentrations. The observed changes in cortisol concentrations represent mild stress responses, as they are only a fraction of the range of the regular circadian changes in cortisol levels in rhesus monkeys. They also indicate that combinations of procedures do not necessarily lead to cumulative stress responses. Our results indicate that salivary cortisol levels of rhesus monkeys respond to neurophysiological experimental procedures and, hence, may be used to assess further refinements of such experimental methods.

## Introduction

Phylogenetic closeness to humans and similarities in physiology, neuroanatomy, cognition and social complexity make non-human primates (NHPs) a vital research model for neuroscience research [1,2]. Yet, their highly developed sensory, social and cognitive abilities raise ethical concerns and can create significant challenges in terms of ensuring good husbandry and welfare in captive research environments [3].

Neurophysiological research has taken considerable strides to improve the welfare of NHPs in research environments, for instance, by adopting behavioral management practices such as social housing, monitoring of physiological parameters, behavioral observations, and positive reinforcement training (PRT). Despite improvements made, neurophysiological research still requires procedures which likely cause stress and raise welfare concerns. For instance, neurophysiological research typically involves separation of an animal from its social group during scientific procedures, movement restraints (by use of primate chairs), surgical implants including their regular cleaning, and fluid or food control protocols to boost the animal’s motivation to participate in training and recording sessions. These procedures likely cause some level of stress or discomfort to the animals, but the extent and whether stress responses to the application of multiple procedures accumulate, remains unclear.

To improve animal welfare in the research context, the welfare situation needs to be assessed, for example, by identifying stressors that are related to experimental procedures and quantifying associated stress responses. The hypothalamic pituitary adrenal (HPA) axis is the central stress response system, with its activation resulting in the production of cortisol, i.e., the primary glucocorticoid in humans and NHPs [4]. For example, plasma cortisol levels in rhesus macaques (*Macaca mulatta*) have been reported to increase due to social separation [5] and movement restraint [6]. Training monkeys to participate in experimental procedures with positive reinforcement training (PRT), as used widely in system neuroscience, can reduce stress responses, as, for instance, shown for venipuncture [7], entering a transport box [8], and presenting the hind leg for injections [9].

The assessment of cortisol in saliva is a widely accepted and frequently used method to evaluate stress response of an individual [10,11]. Salivary cortisol reflects the unbounded biologically active fraction of that hormone which enters the oral cavity mainly by passive diffusion [10,11]. Concentrations of cortisol in saliva are positively correlated with free cortisol values in blood, mirroring its rise and decline, respectively, with a two- to three-minute lag [12]. This tight correlation makes saliva sampling a suitable approach, avoiding the invasive and hence potentially stressful aspects of plasma sampling. NHPs can be trained to voluntary chew on a dental rope or lick on gauze covering a screen, facilitating repeated sampling within a short time period [13,14].

Here we investigated the effects of routinely used experimental procedures in systems neuroscience, namely, controlled fluid access, cleaning of chronic implants, and training behavioral tasks while being separated from the group and seated in a primate chair, on salivary cortisol levels in behaviorally trained male rhesus macaques in captivity. Based on a previous study that observed no adverse effects of fluid control on blood or renal physiology [15], we predict fluid control to induce no or only short-term HPA responses, which do not lead to lasting health effects. No published data exist on the effect of the regular cleaning of chronic implants in rhesus macaque. We expect some degree of stress and/or discomfort to the animals, because the cleaning procedure involves approaching the monkey from behind, touching its head (a procedure not naturally occurring), and the removal of granulation tissue. Because our animals are extensively accustomed to be seated in a primate chair and to head immobilization, we anticipate the stress response to training sessions to be mild. On the other hand, stress induced by the dependency of the reward on proper performance during training could depend on individual frustration tolerance.

## Materials and methods

### Animals and husbandry

We conducted the study on 16 laboratory-housed male rhesus macaques (4–11 years old; mean = 8.12 years) participating in neurophysiological studies. The animals were housed in facilities of the German Primate Center in Goettingen in isosexual pairs or groups of three to four individuals with visual and auditory contact to other macaque groups. Our facilities’ housing is furnished with fixed and moveable perches, environmental enrichment (e.g., balls, puzzle tubes; see also [16]), and carpeted with wood shavings. The space exceeds the size requirements of the relevant European regulations (3.8 m^3^/2 macaques, EU directive 2010/63/EU). Animals receive monkey chow ad libitum and are supplemented with dried fruits. On days outside the fluid control protocol (see below) they also get fresh fruits and vegetables. The facility has a 12 h light/dark cycle from 7:00 AM to 7:00 PM.

### Neurophysiological research methods

Dietary control protocols, surgical implants and their maintenance, and training sessions in primate chairs are commonly used neurophysiological research methods (see also Table 1). In our laboratory, we train rhesus macaques to perform complex behavioral tasks using operant conditioning in which an attractive stimulus (a liquid reward) serves as an incentive for the animals to participate in the experiments and increase the number of experimental trials [17,18]. To increase the subjective value of this reinforcing signal we employ a commonly used fluid control protocol. Here the animals have unrestricted access to food and fluid, except on the days where data are collected or the animal is trained on the behavioral paradigm. On these days the animals are allowed unlimited access to fluid through their performance in the behavioral paradigm, where they receive fluid rewards for every correctly performed trial. Throughout the animals’ psychological and veterinary well being is monitored by the veterinarians, the animal facility staff and the lab’s scientists, all specialized on working with non-human primates.

**Table 1:** Definitions of experimental conditions and time points of sample collection.

Condition	Definition
Access to fluid free	Within the last 24 h, the animals had free access to fluid for at least 4 h, typically much more.
Access to fluid controlled	Within the last 24 h the fluid control protocol was used, i.e. the animal had access to fluid only through performing a trained behavioral task.
Implant cleaning before vs. after cleaning (*aka* before training)	Cleaning of chronic head implants, by means of removal of dead tissue and application of disinfecting substance. For the duration of cleaning (45–60 min) animals are movement restrained while seated in a primate chair. Samples were collected immediately before starting the cleaning procedure or after completion of the cleaning procedure.
Training session	Animals are given the opportunity to work on a behavioral task for performance-based rewards while fluid access is controlled outside the training sessions. For the duration of training (1–4 h) animals are movement restrained because they are seated in a primate chair. Samples were collected 15 min after completion of the training session.

In addition to applying fluid control, neurophysiological research commonly requires phasic fixation of the monkey’s head via skull-mounted chronic implants (‘head-post’) during recording sessions. This procedure is necessary to prevent mechanical perturbations of the microelectrode recordings, which would be detrimental to the signal quality. In addition to the head post, electrophysiological recordings require a permanent access point (craniotomy with a skull-mounted ‘recording chamber’) to the brain region of interest. Outside of recording sessions this recording chamber is sealed with a plug, preventing contamination of the craniotomy. During recording sessions, the seal is removed, providing access to the dura mater for cleaning or the penetration with microelectrodes for recording purposes. Both types of chronic transcutaneous implants require regular cleaning of the surrounding skin margins to avoid infections. The recording chamber requires additional internal cleaning at regular intervals, usually before and after recording sessions.

Training sessions, in which the animal is seated in a primate chair and placed in a dimmed or darkened room with no visual, auditory or olfactory access to conspecifics, might be an additional source of stress for rhesus macaques. Apart from the movement restraint and the social isolation, training sessions include potential frustrating events for the animal, e.g., if the animals fails to successfully complete individual or series of behavioral tasks.

### Sample collection

**Fig 1:**
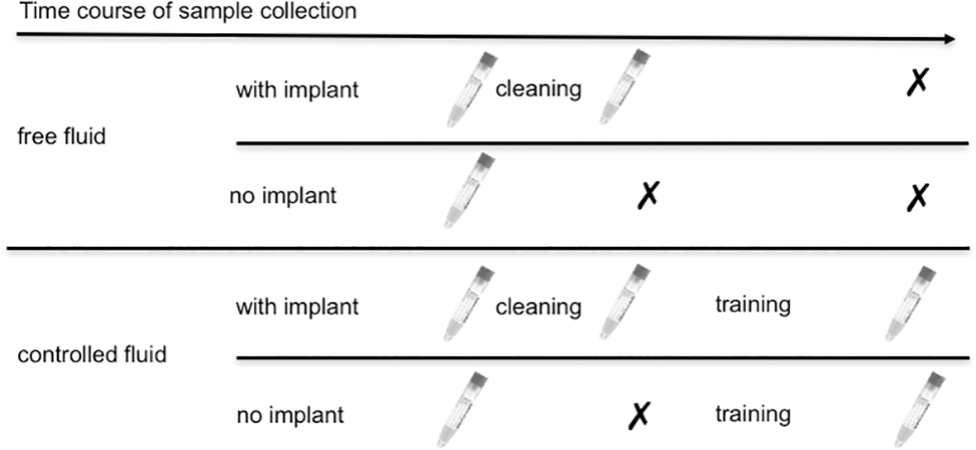
Time points of possible sample collection. Schematic view of samples that potentially could have been collected over the time course of the day depending on the animals’ implantation and training status.

On a given day, rhesus macaques in our laboratory may undergo different procedures (i.e., implant cleaning, training or recording session) depending on their implant and training status. Procedures applied determine whether saliva samples could be taken in the ‘after cleaning’ or ‘after training’ condition. The ‘after cleaning’ condition, for instance, only exists for animals with a chronic implant, and the ‘after training’ condition only exists for animals undergoing behavioral training sessions. Fig 1 graphically summarizes which samples can be taken in which situation.

We collected a total of 767 saliva samples either before implant cleaning, after implant cleaning, or after prolonged sessions of training to perform behavioral tasks. All samples reported here were collected while the animal was seated in a primate chair.

We also collected saliva samples while monkeys were in their housing facility. These samples, however, were often contaminated with other material, e.g. dirt, fecal matter or urine, and, hence, had to be discarded.

Measuring an animal’s stress response in relation to the different experimental treatments requires sample collection itself to be stress-free. The animals were trained to voluntarily chew on cotton swabs (Salivette^®^ from Sarstedt) for at least 45 s and return them on command. Cotton swabs were flavored with sugary juice and dried before use to encourage chewing and adequate salivary volume accumulation in the swab [19]. After collection, saliva samples were stored at -20°C until analysis.

### Hormone analysis

For the analysis, samples were thawed and centrifuged at 3000 rpm for 10 min. Cortisol concentrations were measured using a commercial immunoassay for saliva and serum (Luminescence Immunoassay RE62011, IBL Hamburg). The immunoassay is based on the competition between the unknown amount of antigen in the sample (here cortisol in saliva) and the fixed amount of enzyme-labeled antigen in the assay. Both antigens compete for binding sites on the antibodies coated in the wells of a hormone concentration measurement plate. After incubation, the competitive reaction is stopped and luminescence substrate solution is added to highlight the amount of enzyme labeled antigen. The intensity of the luminescence measured (Luminometer CentrolLIA LB 961, Berthold Technologies) is inversely proportional to the amount of antigen in the sample. We diluted all standards according to the manufacturer’s specifications for salivary cortisol and accepted only test values of plates whose standards and kit controls fell within the acceptable range stated on the manufacturer’s quality control (QC) certificate. Intra- and inter-assay coefficients of variation for the used assay were 7.1% (N = 20) and 10.7% (N = 10), respectively, and the detectible cortisol values ranged between 0.06–40 ng/ml.

### Data processing

From the 767 samples collected, we excluded 20 (2.61%) samples from the analysis, because either the cortisol concentration of these samples was outside the detectable range (0.06–40 ng/ml), the samples were contaminated with blood, dirt or food matter, or the animals had chewed less than 45 s on the cotton swabs. Additionally, we discarded 6 (0.80%) samples taken 1–31 days after a surgery to ensure that the locomotor function, an indicator for physical health [20], had returned to baseline.

Prior to statistical analysis, we checked the distributions of all predictors and the response (i.e., cortisol concentration). We used the natural logarithm to transform the cortisol values (response variable) to achieve a more symmetrical distribution that was normally distributed with homogeneous residuals (verified by visual inspection of a quantile-quantile plot of the residuals and residuals plotted against fitted values).

**Fig 2:**
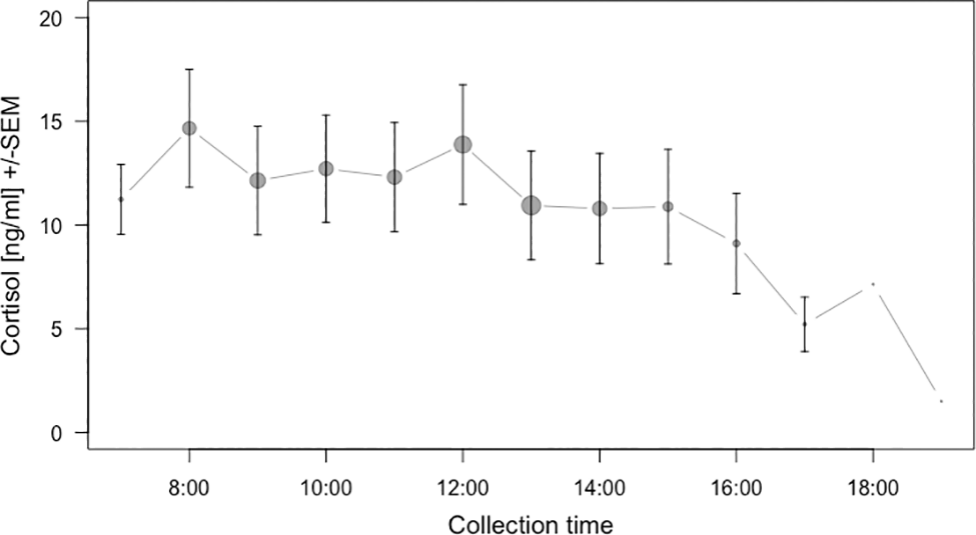
Collection time. Decline of salivary cortisol concentration [ng/ml] over the day (binned for 60 minutes). The typically observed pronounced after-awakening peak and associated circadian decline during the morning hours [11,12] does not show prominently in our data, likely because we collected samples after the animals were awake for at least one hour (at or after 8 am).

When investigating changes in cortisol concentration, the hormone’s circadian rhythm has to be taken into account. In blood and saliva cortisol levels follow a pronounced circadian rhythm, with a peak about 30 minutes after awakening in the morning, followed by a steady decline over the day [11,12]. There can be secondary peaks related to larger meals [12]. Adult male rhesus macaques, have been reported to show a 220% higher cortisol excretion in the morning than the afternoon [21]. In our dataset, cortisol concentrations also followed a circadian rhythm, with the concentrations in the morning being about 160% higher than in the late afternoon (Fig 2). To account for the circadian rhythm, we included the continuous predictor ‘collection time’ into our model. Collection time was mean-centered to achieve comparable model estimates.

### Statistical analyses

To account for the circadian rhythm of cortisol and a potentially non-linear effect of collection time we fitted all models using a Generalized Additive Mixed Model (GAMM) with Gaussian error structure and identity link function (i.e., linear predictor equals fitted value). The GAMM is a generalized linear mixed model in which the linear form of the predictor(s) is replaced by an unspecified smooth function [22]. In our analyses the smooth function accounts for the variation in cortisol samples from various time points across the day. We fitted the data in R v.3.0.1 [23] using the function “gamm” provided by the package “mgcv” [24]. After fitting each model, we checked the effect structure of collection time on cortisol concentration. This effect turned out to be linear with only one effective degree of freedom, documenting the known continuous drop in cortisol concentration from morning to evening. In all fitted models, we accounted for inter-individual variability by including animal identity as random effect. Additionally, we checked all models for stability by excluding data points one by one from the dataset and comparing the estimates derived with those obtained for the full model; we found no indication of influential cases.

We first tested whether animals with a chronic implant differed in their cortisol concentrations from those without an implant. We only used samples taken before cleaning and of animals with free access to fluid to avoid possible effects of implant cleaning and fluid control on the analysis. Since there was no effect of chronic implants on cortisol concentration (see results section), we pooled samples of animals with and without implants for all subsequent analyses.

In order to investigate the effect of the type of access to fluid, implant cleaning, and training sessions on the cortisol concentration, we created two datasets. This division was necessary, because for training sessions animals were always under fluid control. We therefore needed to limit the two ‘before training’ conditions (i.e., before cleaning and after cleaning) to those samples collected when the animal was under fluid control. First, we tested the effect of type of access to fluid and implant cleaning on cortisol concentration, incorporating the possible interaction between these two parameters into the model. Subsequently, we ran a separate model investigating the effect of training session on cortisol concentration, i.e., investigating the change in cortisol concentration between samples taken before and after cleaning (when under controlled access to fluid) with those taken after training. Table 2 indicates the number of samples and animals for all test conditions, with a box indicating the samples used in the model testing the effect of implant cleaning and fluid control, and a grey background for the model testing the effect of training on salivary cortisol concentration.

**Table 2:** Overview of collected samples.

sample collection		before implant cleaning	after implant cleaning	after training
**free fluid**	**with implant**	N_subjects_ = 7 N_samples_ = 78	N_subjects_ = 7 N_samples_ = 108	NA
	**no implant**	N_subjects_ = 6 N_samples_ = 97	NA	NA
**controlled fluid**	**with implant**	N_subjects_ = 7 N_samples_ = 154	N_subjects_ = 7 N_samples_ = 25	N_subjects_ = 2 N_samples_ = 29
	**no implant**	N_subjects_ = 6 N_samples_ = 145	NA	N_subjects_ = 4 N_samples_ = 105

Number of animals and samples collected with free vs. controlled access to fluid, with and without implant, as well as before implant cleaning, after implant cleaning, and after training. Since carrying chronic implant did not affect cortisol level, animals with and without implants were pooled for subsequent analyses. The box indicates samples used to investigate the effect of implant cleaning and access to water on cortisol concentration, and the dark-grey background indicates samples used to analyze the effect of training on cortisol concentration.

### Ethics statement

Research with non-human primates represents a small but indispensable component of neuroscience research. The scientists in this study are aware and are committed to the great responsibility they have in ensuring the best possible science with the least possible harm to the animals [25]. All animal procedures were conducted according to the relevant national and international laws and guidelines, including the German Animal Protection Law, the European Union Directive 2010/63/EU on the Protection of Animals used for Scientific Purposes and the Society for Neuroscience Policies on the Use of Animals and Humans in Neuroscience Research. All animal procedures have been approved by the responsible regional government office (Niedersaechsisches Landesamt fuer Verbraucherschutz und Lebensmittelsicherheit (LAVES)) under the permit numbers 33.14.42502-04-064/07 and 3392 42502-04-13/1100. As all data collected for this study came from animals participating in other studies no additional animals were needed for this study.

## Results

In this study we assessed saliva cortisol concentrations for male rhesus monkeys with vs. without implants and in five conditions. We first tested whether the animals with a chronic implant differed in their cortisol concentrations from those without an implant. We only used samples taken before cleaning and of animals with free access to fluid (total N = 175; 78 from 7 animals with an implant, 97 from 6 animals without an implant) to avoid possible effects of implant cleaning and fluid control on the analysis. Cortisol concentrations did not differ for the two animal groups (GAMM: F_2,11_ = -0.03, P = 0.98). For all subsequent analyses, we therefore did not differentiate between samples from animals with and without implants.

**Fig 3:**
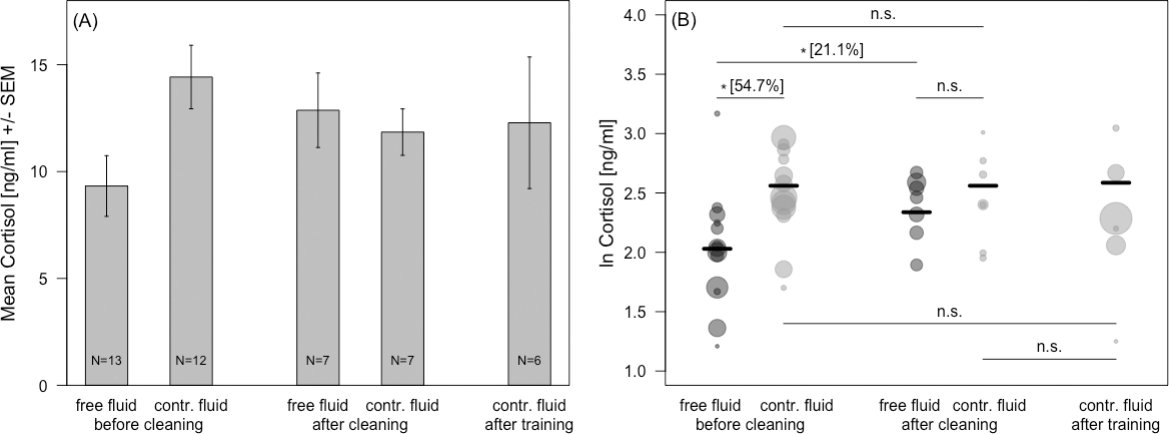
Cortisol values for applied fluid protocol, implant cleaning, and training session. (A) Mean ± standard error (SEM) of cortisol concentrations averaged across the mean values of each animal for a given condition. N = number of animals contributing to the value. (B) Cortisol concentrations (ln-transformed) of saliva samples collected across conditions as used in the GAMM. Each circle depicts the mean value of ln-transformed cortisol values per individual, with the size of the circles indicating the number of samples for that data point. Horizontal black bars indicate the predicted values for cortisol from the model. To aid interpretation of displayed model estimates, collection times were z-transformed. See also Tables 2 and 3, respectively. * indicate p < 0.0001, n.s. represent p > 0.05. Percentages shown represent magnitudes of significant increases in mean cortisol values plotted in (A).

The five conditions we investigated were: either before implant cleaning (for animals with free access to fluid or with controlled access), after implant cleaning (again for two fluid access conditions), or after prolonged sessions of training to perform behavioral tasks (for animals with controlled access to fluid). The corresponding cortisol concentrations are shown in Fig 3A.

Mean individual saliva cortisol concentrations showed substantial variability, combining inter-animal and inter-condition effects, covering an approx. 7-fold range from 3.35 to 24.84 ng/ml, with the highest value in the *after training* condition and the lowest value in the condition before cleaning with free access to fluid. Before cleaning, the average cortisol concentration across the means for every animal was 9.32 ng/ml ± 1.42, SD = 5.12 (mean ± standard error of the mean, SD = standard deviation) with free access to fluid, and 14.42 ng/ml ± 1.48, 5.14 with controlled fluid access. After cleaning, it was 12.87 ng/ml ± 1.72, 4.61 with free access to fluid, and 11.84 ng/ml ± 1.08, 2.87 with controlled access. After training sessions the value was 12.28 ng/ml ± 3.08, 7.54.

To account for potential circadian modulations in cortisol level and avoid corresponding confounds of sampling time, we ran a Generalized Additive Mixed Model with Gaussian error structure and identity link function (see Methods). The left-hand and middle panels in Fig 3B depict model estimates of cortisol concentrations (*ln*-transformed) in the condition before vs. after cleaning with free vs. controlled access to fluid. Each circle indicates mean values per individual, with the circle size referring to the number of samples for that data point. Generally, model estimates for cortisol concentrations were higher when the animal was under fluid control compared to free access to fluid, and when the sample was taken after implant cleaning compared to before (Fig 3B, horizontal bars). We found a significant interaction between access to fluid and implant cleaning on cortisol concentration (GAMM: F_4,588_ = 4.23, P = 0.0024, Table 3), with samples collected before cleaning showing a significant lower concentration of cortisol when the animal had free access to fluid, compared to when the animal was under fluid control (F_4,588_ = 8.58, P < 0.001, 54.7% higher cortisol concentration with controlled vs. free access to fluid). Samples collected after implant cleaning showed no difference in their cortisol levels as function of access to fluid (F_4,588_ = -1.22, P = 0.22). Implant cleaning had an effect on cortisol concentration when the animal had free access to fluid, with cortisol values before cleaning significantly lower than cortisol values after cleaning (F_4,588_ = -4.23, P < 0.001, 21.1% higher cortisol concentration after cleaning compared to before cleaning). Rhesus macaques under fluid control showed no effect of implant cleaning (F_4,588_ = 0.79, P = 0.42). Over the time of the day the cortisol concentration decreased, by a factor of 0.052 times per hour (F_4,588_ = -5.67, P < 0.001, Table 3).

**Table 3:** Effect of implant cleaning and access to fluid on salivary cortisol.

Predictor variable	Estimate	SE	DF	F	p	lower CI	upper CI
Intercept^1^ (after cleaning, free fluid)	2.34	0.08	588	27.82	<0.0001	2.17	2.50
Intercept^2^ (bef. cleaning, contr. fluid)	2.56	0.07	588	35.30	<0.0001	2.42	2.70
Implant cleaning							
before vs. after (fluid free)^1^	-0.31	0.07	588	-4.24	<0.0001	-0.45	-0.17
after vs. before (contr. fluid)^2^	-0.09	0.11	588	-0.80	0.427	-0.29	0.12
Access to fluid							
controlled vs. free (after cleaning)^1^	0.14	0.12	588	1.22	0.222	-0.09	0.36
free vs. controlled (bef. cleaning)^2^	-0.53	0.06	588	-8.59	<0.0001	-0.65	-0.41
Implant cleaning:access to fluid							
before:controlled vs. after:free^1^	0.39	0.13	588	3.05	0.002	0.14	0.65
after:free vs. before:controlled^2^	0.39	0.13	588	3.05	0.002	0.141	0.65
Collection time^1^^&^^2^	-0.06	0.01	588	-5.67	<0.0001	-0.074	-0.04

Results of the Generalized Additive Mixed Model investigating the effect of implant cleaning (before vs. after) and access to fluid (free vs. controlled) on the salivary cortisol concentration of male rhesus macaques. IC = 95% Confidence Interval.

^1&2^ Identifies the intercept to which the conditions are compared to.

Since controlling access to fluid is typically used to boost the motivation of rhesus macaques in training sessions, samples used to explore the effect of training on cortisol concentrations were collected under fluid control. There was no difference in cortisol concentration before training (i.e., before cleaning, after cleaning) and after training observed (before cleaning vs. after training: F_2,443_ = -1.51, P = 0.13, after cleaning vs. after training: F_2,443_ = -0.95, P = 0.34, Fig 3B and Table 4). Similar to our previous model, a sample which was collected one hour later resulted on average in a 0.05 times lower cortisol concentration (F_2,443_ = -3.96, P < 0.001, Table 4).

**Table 4:** Effect of chaired training sessions on salivary cortisol.

Predictor variable	Estimate	SE	DF	F	p	lower CI	upper CI
Intercept	2.59	0.12	443	22.39	<0.0001	2.36	2.81
Implant cleaning							
before	-0.10	0.07	443	-1.51	0.132	-0.23	0.03
after	-0.12	0.12	443	-0.96	0.340	-0.35	0.12
Collection time	-0.05	0.02	443	-3.96	<0.0001	-0.07	-0.02

Results of the Generalized Additive Mixed Model investigating the effect of training session (before vs. after) on the salivary cortisol concentration of male rhesus macaques. Before and after cleaning corresponds to the before training condition. All samples were collected when the animal under examination was under fluid control. The level ‘after training’ is indicated in the intercept. IC = 95% Confidence Interval.

## Discussion

In neurophysiological research NHPs (and other animals) are subject of routine handling, veterinary and experimental procedures. Despite animals being accustomed to the procedures using PRT they may still elicit physiological stress responses (e.g., [26–28]). Here we examined the effect of routinely applied fluid access protocols (free vs. controlled access), the presence of chronic implants and their cleaning, as well as physical restraint in the primate chair during behavioral training sessions on rhesus macaque males. We expected, observed and accounted for the decline in cortisol concentration, with saliva samples taken earlier in the day containing 160% higher cortisol concentrations than samples taken in the afternoon. The circadian decline is in line with the morning-afternoon modulation reported in previous studies [11,12].

Cortisol levels in animals with and without chronic implants did not differ. Both, controlled fluid regime and cleaning of implants increased salivary cortisol levels, but both together did not further increase the level. Specifically, before cleaning, rhesus macaques under fluid control had 54.7% higher cortisol concentration than individuals with free access to fluid. Under free access to fluid, animals showed an effect of cleaning, with 27.1% higher cortisol concentrations after vs. before implant cleaning. After cleaning, cortisol levels in monkeys with controlled vs. free access to fluid were not significantly different. Also, animals under fluid control showed no difference between before and after cleaning, or between before and after training. This lacking further increase in cortisol in response to implant cleaning or training of fluid-controlled animals is unlikely to be due to a lack in statistical power since there is not even a trend towards higher values compared to the values before implant cleaning (Fig 3A).

The observed moderate increases in cortisol concentration can be considered mild stress responses. First, the effects of experimental conditions on cortisol concentration we observed fell in the lower range of reported effects other stressors cause on cortisol. For instance, the relocation of rhesus macaques to a new and unknown environment without prior training, resulted in an increase in cortisol concentration ranging between 166.0% [29] and 180.0% [6]. Venipuncture of trained female rhesus macaques elicited a 31.8% increase in cortisol concentration, compared to baseline values [9], with trained animals showing 51.8% lower baseline cortisol concentrations than untrained animals [9]. Exposure to noise, such as construction work underneath the housing facility, also caused elevated stress response, i.e., 50% higher cortisol concentrations with construction noise vs. without noise (long-tailed macaques, *M*. *fascicularis* [30]. The separation of rhesus macaque infants from their mothers for 40 min [31] or 3 h [32] lead to a mean increase in cortisol concentrations of about 22.4% and 40.0%, respectively. Second, the magnitude of the changes we observed are within a fraction of the range of cortisol level changes within the regular circadian rhythm in healthy animals. For comparison, adult male rhesus macaques show a circadian cortisol level modulation, with the maximum salivary cortisol in the morning hours, about 220% higher than the values in the late afternoon [21].

Cortisol levels did not add for combinations of procedures, indicating no accumulation of stress responses. We did not observe an additive effect between controlled access to fluid and implant cleaning or training, respectively. This lack of additivity cannot be explained by a ceiling effect. For example, the high circadian fluctuation in cortisol level (160% this study, 220% [21]) compared with the changes in cortisol concentration found in our study (54.7% effect of fluid access and 21.1% effect of cleaning, with free access to fluid, respectively) are evidence that changes in cortisol concentration could easily have been higher. Given the level of overall increases and the lack of additivity, we conclude that the experimental procedures investigated here cause only mild stress responses in our rhesus macaques. Our extensive training on these procedures might contribute to this.

The way sample were collected and experimental procedures applied did not interfere with the cortisol measurement itself. One might argue that controlling the monkeys’ access to fluid affects their saliva flow rate and, subsequently, affects the measured salivary cortisol concentration. However, neither the increase of saliva flow by administration of citric acid to the tongue nor its decrease with anticholinergic drugs (‘dry mouth’) significantly influenced the salivary cortisol concentration in previous studies (see [33] and references therein). This is due to the small size and lipophilic nature of cortisol (like any other steroid) that enables its unbounded, and biologically relevant, fraction to rapidly diffuse through the lipid-rich cell membranes from the blood to salivary glands into saliva. Therefore, we rule out that the effect of the fluid protocol (free vs controlled) on salivary cortisol is a side-effect of changes in saliva concentration, but rather is indicative of a physiological HPA response.

Repeated short-term stress responses do not necessarily lead to long-term consequences. Neither blood values nor kidney controls indicate any lasting consequences, such as kidney dysfunction, even after years of routinely applied fluid protocols [15]. This suggests that while causing mild short-term stress responses, the variable availability of fluid does not overtax regular physiological compensation mechanisms. Further research will have to show if alternative methods exist that successfully boost an animal’s motivation to participate in behavioral tasks and elicit even smaller or no stress responses.

The duration of physical constraint in the primate chair and the behavioral training on cognitive tasks did not increase cortisol responses. While rhesus macaque males that were subject to fluid control or whose implants were cleaned showed elevated levels of salivary cortisol, we found similar cortisol values before and after training sessions. This indicates that training, which includes social separation and movement constraints due to chair seating for significantly longer durations than just implant cleaning, does not lead to a further increase in cortisol concentrations. While previous studies exist in rhesus monkeys that tested stress responses to immobilization and physical restrain in various contexts [34], including short-term habituation to primate chairs [28], we are not aware of data on the effect of routine chair-seating in the context of extensive and systematic positive reinforcement training in monkeys. Our results show that, once seated in the primate chair, neither the additional time per se that the animals spent in the chair to train on a behavioral task separate from the peer group, nor the potential frustration from performance-contingent reward schedules induced a further stress response. To test the sole effect of chair-seated training, one would need to sample animals that are trained without fluid control.

It is important to keep in mind that the brain, behavior, hormones and immune system are interdependent, and that a disturbance in one typically affects one or all of the others [35]. Thus the welfare of research animals affects the quality of scientific data and their interpretation and, hence, needs to be optimized [35,36]. With salivary cortisol measures we were able to quantify mild stress responses to the routine procedures used here and in many other systems neuroscience studies working with non-human primates. This means that salivary cortisol is sensitive to detect changes in HPA activity of rhesus monkeys in neurophysiological experimental settings and, hence, can be used to evaluate the level of stress within these settings as well as potential refinements of the methods.

## Supporting information

S1 Dataset Raw data to perform statistical analysis. Cortisol values per animal, condition, collection time, and date.
(PDF)

S1 Table Summary of cortisol values per animal and condition. Mean cortisol values (ln-transformed) per animal and condition.
(PDF)
