# Supplementary material for: Assessment of stress responses in rhesus macaques (Macaca mulatta) to daily routine procedures in system neuroscience based on salivary cortisol concentrations
Source: PLoS One. 2018 Jan 2;13(1):e0190190. doi: 10.1371/journal.pone.0190190 (PMC5749769; doi:10.1371/journal.pone.0190190)
Supplement: S1 Dataset — Cortisol values per animal, condition, collection time, and date. (PDF) [file pone.0190190.s001.pdf]

**S1 Dataset: Raw data to perform statistical analysis.** Cortisol values per animal, condition, collection time, and date.

| date [ddmmyy] | collect_time [min] | animal_ID | chamber_on | Cortisol [ng_ml] | condition | fluid |
|---|---|---|---|---|---|---|
| 02.06.08 | 570 | Pie | yes | 16.92 | after_cleaning | controlled |
| 02.06.08 | 585 | Syl | yes | 20.95 | before_cleaning | controlled |
| 02.06.08 | 600 | Sun | no | 9.91 | before_cleaning | controlled |
| 03.06.08 | 505 | Car | no | 20.85 | before_cleaning | controlled |
| 03.06.08 | 530 | Sun | no | 11.42 | before_cleaning | controlled |
| 03.06.08 | 640 | Syl | yes | 11.19 | before_cleaning | controlled |
| 03.06.08 | 720 | Gra | no | 19 | before_cleaning | controlled |
| 04.06.08 | 495 | Car | no | 18.6 | before_cleaning | controlled |
| 04.06.08 | 625 | Sun | no | 13.12 | before_cleaning | controlled |
| 05.06.08 | 500 | Mar | no | 7.51 | before_cleaning | controlled |
| 05.06.08 | 525 | Car | no | 11.93 | before_cleaning | controlled |
| 05.06.08 | 880 | Syl | yes | 12.94 | before_cleaning | controlled |
| 09.06.08 | 510 | Wal | yes | 12.33 | before_cleaning | free |
| 09.06.08 | 885 | Tin | yes | 22.28 | before_cleaning | controlled |
| 09.06.08 | 940 | Syl | yes | 5.4 | before_cleaning | free |
| 10.06.08 | 540 | Sun | no | 13.11 | before_cleaning | controlled |
| 10.06.08 | 790 | Wal | yes | 7.17 | before_cleaning | free |
| 10.06.08 | 810 | Syl | yes | 6.97 | before_cleaning | free |
| 10.06.08 | 860 | Tin | yes | 14.77 | before_cleaning | controlled |
| 11.06.08 | 510 | Wal | yes | 12.03 | before_cleaning | free |
| 11.06.08 | 640 | Tin | yes | 19.27 | before_cleaning | controlled |
| 11.06.08 | 945 | Syl | yes | 5.21 | before_cleaning | free |
| 12.06.08 | 690 | Sun | no | 15.5 | before_cleaning | controlled |
| 12.06.08 | 800 | Tin | yes | 21.49 | before_cleaning | controlled |
| 12.06.08 | 850 | Syl | yes | 9.98 | before_cleaning | free |
| 13.06.08 | 685 | Sun | no | 23.7 | before_cleaning | controlled |
| 13.06.08 | 725 | Tin | yes | 31.26 | before_cleaning | controlled |
| 13.06.08 | 960 | Syl | yes | 10.55 | before_cleaning | free |
| 14.06.08 | 565 | Wal | yes | 8.63 | before_cleaning | free |
| 16.06.08 | 670 | Pie | yes | 22.4 | before_cleaning | controlled |
| 16.06.08 | 855 | Syl | yes | 15.66 | before_cleaning | free |
| 17.06.08 | 510 | Wal | yes | 6.37 | before_cleaning | controlled |
| 17.06.08 | 555 | Pie | yes | 17.86 | before_cleaning | controlled |
| 17.06.08 | 570 | Sun | no | 11.7 | before_cleaning | controlled |
| 17.06.08 | 810 | Syl | yes | 12.52 | before_cleaning | controlled |
| 18.06.08 | 525 | Mar | no | 27.26 | before_cleaning | controlled |
| 18.06.08 | 535 | Wal | yes | 6.17 | before_cleaning | controlled |
| 18.06.08 | 970 | Syl | yes | 15.57 | before_cleaning | controlled |
| 19.06.08 | 820 | Tin | yes | 25.88 | before_cleaning | controlled |
| 20.06.08 | 560 | Pie | yes | 25.4 | before_cleaning | controlled |
| 20.06.08 | 730 | Syl | yes | 25.2 | before_cleaning | controlled |

| | | | | | | |
|---|---|---|---|---|---|---|
| 21.06.08 | 455 | Wal | yes | 8.57 | before_cleaning | controlled |
| 22.06.08 | 485 | Wal | yes | 9.27 | before_cleaning | controlled |
| 23.06.08 | 510 | Wal | yes | 11.19 | before_cleaning | controlled |
| 23.06.08 | 630 | Tin | yes | 13.11 | before_cleaning | controlled |
| 23.06.08 | 645 | Syl | yes | 15.38 | before_cleaning | controlled |
| 23.06.08 | 820 | Sun | no | 13.38 | before_cleaning | controlled |
| 24.06.08 | 565 | Tin | yes | 23.97 | before_cleaning | controlled |
| 24.06.08 | 635 | Sun | no | 17.27 | before_cleaning | controlled |
| 24.06.08 | 810 | Mar | no | 8.6 | before_cleaning | controlled |
| 24.06.08 | 815 | Pie | yes | 8.13 | before_cleaning | controlled |
| 24.06.08 | 860 | Syl | yes | 12.48 | before_cleaning | controlled |
| 25.06.08 | 525 | Sun | no | 27.6 | before_cleaning | controlled |
| 25.06.08 | 630 | Pie | yes | 13.53 | before_cleaning | controlled |
| 25.06.08 | 650 | Syl | yes | 14.04 | before_cleaning | controlled |
| 25.06.08 | 830 | Tin | yes | 16.13 | before_cleaning | controlled |
| 26.06.08 | 555 | Sun | no | 19.02 | before_cleaning | controlled |
| 26.06.08 | 835 | Tin | yes | 15.75 | before_cleaning | controlled |
| 30.06.08 | 555 | Tin | yes | 23.01 | before_cleaning | controlled |
| 30.06.08 | 900 | Syl | yes | 11.78 | before_cleaning | controlled |
| 30.06.08 | 930 | Mar | no | 5.11 | before_cleaning | controlled |
| 01.07.08 | 670 | Syl | yes | 8.1 | before_cleaning | controlled |
| 01.07.08 | 795 | Sun | no | 10.4 | before_cleaning | controlled |
| 01.07.08 | 810 | Wal | yes | 6.19 | before_cleaning | controlled |
| 02.07.08 | 540 | Mar | no | 11.27 | before_cleaning | controlled |
| 02.07.08 | 705 | Sun | no | 24.3 | before_cleaning | controlled |
| 02.07.08 | 815 | Syl | yes | 37.55 | before_cleaning | controlled |
| 03.07.08 | 570 | Mar | no | 10.9 | before_cleaning | controlled |
| 04.07.08 | 720 | Hay | yes | 8.34 | before_cleaning | free |
| 04.07.08 | 793 | Sun | no | 26.44 | before_cleaning | controlled |
| 04.07.08 | 910 | Syl | yes | 10.43 | before_cleaning | controlled |
| 07.07.08 | 510 | Wal | yes | 6.78 | before_cleaning | controlled |
| 07.07.08 | 535 | Pie | yes | 11.23 | before_cleaning | controlled |
| 07.07.08 | 540 | Mar | no | 5.64 | before_cleaning | controlled |
| 07.07.08 | 645 | Syl | yes | 15.23 | before_cleaning | controlled |
| 07.07.08 | 795 | Tin | yes | 15.08 | before_cleaning | controlled |
| 08.07.08 | 510 | Wal | yes | 9 | before_cleaning | controlled |
| 08.07.08 | 510 | Mar | no | 26.45 | before_cleaning | controlled |
| 08.07.08 | 765 | Syl | yes | 18.71 | before_cleaning | controlled |
| 09.07.08 | 510 | Wal | yes | 6.42 | before_cleaning | controlled |
| 09.07.08 | 665 | Pie | yes | 14.93 | after_cleaning | controlled |
| 09.07.08 | 840 | Tin | yes | 12.54 | before_cleaning | controlled |
| 10.07.08 | 540 | Mar | no | 15.7 | before_cleaning | controlled |
| 10.07.08 | 640 | Syl | yes | 18.35 | before_cleaning | controlled |
| 10.07.08 | 835 | Tin | yes | 24.94 | before_cleaning | controlled |
| 11.07.08 | 555 | Mar | no | 13.98 | before_cleaning | controlled |

| | | | | | | |
|---|---|---|---|---|---|---|
| 11.07.08 | 855 | Syl | yes | 12.72 | before_cleaning | controlled |
| 12.07.08 | 465 | Tin | yes | 13.7 | before_cleaning | controlled |
| 13.07.08 | 545 | Tin | yes | 26.4 | before_cleaning | controlled |
| 14.07.08 | 500 | Syl | yes | 16.6 | before_cleaning | controlled |
| 15.07.08 | 555 | Pie | yes | 18.43 | before_cleaning | controlled |
| 15.07.08 | 680 | Car | yes | 6.88 | before_cleaning | free |
| 15.07.08 | 750 | Sun | no | 9.2 | before_cleaning | controlled |
| 15.07.08 | 825 | Mar | no | 8 | before_cleaning | controlled |
| 15.07.08 | 840 | Syl | yes | 13.85 | before_cleaning | controlled |
| 16.07.08 | 525 | Hay | yes | 12 | before_cleaning | free |
| 16.07.08 | 580 | Car | yes | 8.67 | before_cleaning | free |
| 16.07.08 | 640 | Tin | yes | 32.92 | before_cleaning | controlled |
| 17.07.08 | 540 | Hay | yes | 34.24 | before_cleaning | free |
| 17.07.08 | 590 | Car | yes | 6.6 | before_cleaning | free |
| 17.07.08 | 720 | Wal | yes | 5.46 | before_cleaning | controlled |
| 18.07.08 | 565 | Car | yes | 11.19 | before_cleaning | free |
| 18.07.08 | 590 | Hay | yes | 17.99 | before_cleaning | free |
| 18.07.08 | 765 | Tin | yes | 13.03 | before_cleaning | controlled |
| 21.07.08 | 580 | Pie | yes | 16.37 | before_cleaning | controlled |
| 21.07.08 | 735 | Mar | no | 9.13 | before_cleaning | controlled |
| 21.07.08 | 780 | Hay | yes | 7.42 | before_cleaning | free |
| 21.07.08 | 915 | Sun | no | 9.1 | before_cleaning | controlled |
| 22.07.08 | 510 | Gra | no | 6.83 | before_cleaning | controlled |
| 22.07.08 | 530 | Pie | yes | 19.25 | before_cleaning | controlled |
| 22.07.08 | 570 | Sun | no | 14.81 | before_cleaning | controlled |
| 22.07.08 | 720 | Fer | no | 11.01 | before_cleaning | controlled |
| 22.07.08 | 785 | Wal | yes | 4.24 | before_cleaning | controlled |
| 22.07.08 | 820 | Car | yes | 6.8 | before_cleaning | controlled |
| 22.07.08 | 840 | Edg | no | 4.71 | before_cleaning | free |
| 23.07.08 | 510 | Gra | no | 29.75 | before_cleaning | controlled |
| 23.07.08 | 720 | Fer | no | 33.75 | before_cleaning | controlled |
| 23.07.08 | 780 | Sun | no | 17.52 | after_training | controlled |
| 23.07.08 | 840 | Syl | yes | 18.35 | before_cleaning | controlled |
| 24.07.08 | 590 | Hay | yes | 5.62 | before_cleaning | free |
| 24.07.08 | 660 | Edg | no | 8.71 | before_cleaning | free |
| 24.07.08 | 735 | Wal | yes | 6.54 | after_training | controlled |
| 24.07.08 | 810 | Syl | yes | 28.3 | before_cleaning | controlled |
| 25.07.08 | 495 | Gra | no | 27.23 | before_cleaning | controlled |
| 25.07.08 | 530 | Hay | yes | 7.83 | before_cleaning | free |
| 25.07.08 | 630 | Car | yes | 8.25 | before_cleaning | controlled |
| 25.07.08 | 660 | Edg | no | 9.88 | before_cleaning | free |
| 25.07.08 | 675 | Sun | no | 26.95 | before_cleaning | controlled |
| 25.07.08 | 850 | Luk | no | 20.99 | before_cleaning | free |
| 28.07.08 | 485 | Edg | no | 6.88 | before_cleaning | free |
| 28.07.08 | 500 | Luk | no | 22.91 | before_cleaning | free |

| | | | | | | |
|---|---|---|---|---|---|---|
| 28.07.08 | 560 | Tin | yes | 12.75 | before_cleaning | controlled |
| 28.07.08 | 725 | Wal | yes | 8.32 | after_training | controlled |
| 28.07.08 | 775 | Hay | yes | 7.77 | before_cleaning | free |
| 28.07.08 | 865 | Pie | yes | 14.67 | before_cleaning | controlled |
| 28.07.08 | 875 | Car | yes | 31.52 | after_cleaning | controlled |
| 28.07.08 | 1050 | Mar | no | 6.6 | before_cleaning | controlled |
| 29.07.08 | 490 | Edg | no | 7.17 | before_cleaning | free |
| 29.07.08 | 510 | Luk | no | 19.12 | before_cleaning | free |
| 29.07.08 | 610 | Mar | no | 6.54 | before_cleaning | controlled |
| 29.07.08 | 570 | Tin | yes | 13.37 | after_cleaning | controlled |
| 30.07.08 | 490 | Edg | no | 12.16 | before_cleaning | free |
| 30.07.08 | 525 | Pie | yes | 35.99 | before_cleaning | controlled |
| 30.07.08 | 590 | Car | yes | 15.54 | before_cleaning | controlled |
| 30.07.08 | 840 | Tin | yes | 12.02 | before_cleaning | controlled |
| 30.07.08 | 870 | Syl | yes | 13.48 | before_cleaning | free |
| 30.07.08 | 890 | Sun | no | 15.46 | after_training | controlled |
| 30.07.08 | 965 | Hay | yes | 4.22 | before_cleaning | free |
| 31.07.08 | 495 | Edg | no | 7.49 | before_cleaning | free |
| 31.07.08 | 510 | Tin | yes | 22.76 | before_cleaning | controlled |
| 31.07.08 | 575 | Mar | no | 9.88 | before_cleaning | controlled |
| 31.07.08 | 810 | Car | yes | 11.95 | after_cleaning | controlled |
| 31.07.08 | 900 | Sun | no | 4.3 | before_cleaning | controlled |
| 01.08.08 | 480 | Edg | no | 8.9 | before_cleaning | free |
| 01.08.08 | 510 | Tin | yes | 17.74 | before_cleaning | controlled |
| 01.08.08 | 570 | Sun | no | 24.52 | before_cleaning | controlled |
| 01.08.08 | 570 | Wal | yes | 5.35 | after_cleaning | controlled |
| 01.08.08 | 815 | Car | yes | 10.51 | after_cleaning | controlled |
| 01.08.08 | 960 | Fer | no | 7.86 | before_cleaning | controlled |
| 01.08.08 | 1180 | Hay | yes | 1.51 | before_cleaning | free |
| 03.08.08 | 480 | Fer | no | 33.81 | before_cleaning | controlled |
| 04.08.08 | 760 | Sun | no | 9.3 | after_training | controlled |
| 04.08.08 | 855 | Tin | yes | 12.35 | before_cleaning | controlled |
| 05.08.08 | 500 | Tin | yes | 11.66 | before_cleaning | controlled |
| 05.08.08 | 735 | Pie | yes | 21.81 | before_cleaning | controlled |
| 05.08.08 | 820 | Car | yes | 9.19 | after_cleaning | controlled |
| 05.08.08 | 850 | Edg | no | 3.92 | before_cleaning | free |
| 05.08.08 | 900 | Sun | no | 6.91 | after_training | controlled |
| 06.08.08 | 680 | Wal | yes | 6.41 | after_training | controlled |
| 06.08.08 | 815 | Car | yes | 7.12 | after_cleaning | controlled |
| 06.08.08 | 825 | Sun | no | 5.03 | after_training | controlled |
| 07.08.08 | 680 | Sun | no | 12.4 | after_training | controlled |
| 07.08.08 | 1010 | Wal | yes | 4.62 | after_training | controlled |
| 08.08.08 | 550 | Hay | yes | 6.74 | before_cleaning | free |
| 08.08.08 | 960 | Wal | yes | 6.43 | after_training | controlled |
| 11.08.08 | 800 | Wal | yes | 6.03 | after_training | controlled |

| | | | | | | |
|---|---|---|---|---|---|---|
| 11.08.08 | 840 | Sun | no | 13.24 | after_training | controlled |
| 12.08.08 | 600 | Fer | no | 34.46 | before_cleaning | controlled |
| 12.08.08 | 765 | Sun | no | 10.88 | after_training | controlled |
| 12.08.08 | 950 | Wal | yes | 6.51 | after_training | controlled |
| 13.08.08 | 820 | Sun | no | 15.12 | after_training | controlled |
| 14.08.08 | 660 | Sun | no | 19.36 | after_training | controlled |
| 14.08.08 | 850 | Car | yes | 5.98 | before_cleaning | controlled |
| 15.08.08 | 440 | Car | yes | 12.99 | before_cleaning | controlled |
| 15.08.08 | 780 | Sun | no | 21.51 | after_training | controlled |
| 18.08.08 | 870 | Sun | no | 20.75 | after_training | controlled |
| 19.08.08 | 730 | Sun | no | 16.75 | after_training | controlled |
| 19.08.08 | 900 | Wal | yes | 10.34 | after_training | controlled |
| 20.08.08 | 695 | Sun | no | 26.87 | after_training | controlled |
| 20.08.08 | 915 | Wal | yes | 8.42 | after_training | controlled |
| 21.08.08 | 720 | Sun | no | 22.25 | after_training | controlled |
| 21.08.08 | 905 | Wal | yes | 5.06 | after_training | controlled |
| 21.08.08 | 930 | Gra | no | 13.88 | before_cleaning | controlled |
| 22.08.08 | 690 | Sun | no | 14.04 | after_training | controlled |
| 25.08.08 | 805 | Hay | yes | 4.29 | before_cleaning | free |
| 26.08.08 | 680 | Sun | no | 28.41 | after_training | controlled |
| 26.08.08 | 910 | Wal | yes | 4.5 | after_training | controlled |
| 29.08.08 | 545 | Gra | no | 4.9 | before_cleaning | controlled |
| 01.09.08 | 470 | Hay | yes | 6.1 | after_cleaning | free |
| 01.09.08 | 870 | Gra | no | 8.55 | after_training | controlled |
| 01.09.08 | 885 | Wal | yes | 7.8 | after_training | controlled |
| 02.09.08 | 940 | Wal | yes | 12.54 | after_training | controlled |
| 03.09.08 | 610 | Sun | no | 10.14 | after_training | controlled |
| 03.09.08 | 860 | Edg | no | 6.66 | before_cleaning | free |
| 03.09.08 | 960 | Wal | yes | 10.65 | after_training | controlled |
| 04.09.08 | 585 | Tin | yes | 16.6 | after_cleaning | free |
| 04.09.08 | 660 | Sun | no | 16.29 | after_training | controlled |
| 04.09.08 | 965 | Wal | yes | 8.09 | after_training | controlled |
| 05.09.08 | 570 | Tin | yes | 4.37 | before_cleaning | free |
| 08.09.08 | 735 | Wal | yes | 9.47 | after_training | controlled |
| 08.09.08 | 855 | Sun | no | 12.92 | after_training | controlled |
| 09.09.08 | 655 | Sun | no | 7.95 | after_training | controlled |
| 10.09.08 | 870 | Sun | no | 6.36 | after_training | controlled |
| 10.09.08 | 1065 | Pie | yes | 3.35 | before_cleaning | free |
| 11.09.08 | 825 | Car | yes | 10.45 | before_cleaning | free |
| 14.09.08 | 810 | Car | yes | 16.2 | before_cleaning | controlled |
| 15.09.08 | 840 | Tin | yes | 10.49 | after_cleaning | free |
| 15.09.08 | 840 | Gra | no | 29.76 | before_cleaning | controlled |
| 15.09.08 | 940 | Wal | yes | 4.6 | after_training | controlled |
| 16.09.08 | 630 | Wal | yes | 5.34 | after_training | controlled |
| 16.09.08 | 835 | Tin | yes | 21.17 | after_cleaning | free |

| | | | | | | |
|---|---|---|---|---|---|---|
| 19.09.08 | 920 | Tin | yes | 19.88 | after_cleaning | free |
| 22.09.08 | 685 | Wal | yes | 7.84 | after_training | controlled |
| 23.09.08 | 630 | Wal | yes | 9.23 | after_training | controlled |
| 24.09.08 | 690 | Fer | no | 23.38 | before_cleaning | controlled |
| 25.09.08 | 900 | Gra | no | 33.23 | after_training | controlled |
| 26.09.08 | 900 | Gra | no | 32.73 | after_training | controlled |
| 29.09.08 | 745 | Fer | no | 21.29 | after_training | controlled |
| 30.09.08 | 990 | Fer | no | 22.53 | after_training | controlled |
| 01.10.08 | 600 | Nic | yes | 9.59 | before_cleaning | free |
| 01.10.08 | 980 | Fer | no | 22.55 | after_training | controlled |
| 02.10.08 | 690 | Fer | no | 33.24 | after_training | controlled |
| 02.10.08 | 725 | Nic | yes | 7.17 | before_cleaning | free |
| 03.10.08 | 535 | Pie | yes | 8.96 | after_cleaning | free |
| 04.10.08 | 530 | Fer | no | 22.39 | before_cleaning | controlled |
| 05.10.08 | 690 | Fer | no | 8.93 | after_training | controlled |
| 07.10.08 | 960 | Fer | no | 11.73 | after_training | controlled |
| 08.10.08 | 1050 | Fer | no | 4.19 | after_training | controlled |
| 09.10.08 | 930 | Fer | no | 10.5 | after_training | controlled |
| 10.10.08 | 960 | Fer | no | 7.83 | after_training | controlled |
| 13.10.08 | 570 | Wal | yes | 5.48 | before_cleaning | free |
| 13.10.08 | 620 | Sun | no | 12.2 | after_training | controlled |
| 13.10.08 | 635 | Fer | no | 3.87 | before_cleaning | controlled |
| 13.10.08 | 645 | Nic | yes | 14.86 | before_cleaning | free |
| 13.10.08 | 870 | Hay | yes | 4.7 | before_cleaning | free |
| 14.10.08 | 585 | Wal | yes | 8.65 | after_cleaning | free |
| 14.10.08 | 615 | Nic | yes | 18.03 | before_cleaning | free |
| 14.10.08 | 900 | Sun | no | 22.4 | after_training | controlled |
| 15.10.08 | 550 | Gra | no | 38.69 | before_cleaning | controlled |
| 15.10.08 | 630 | Wal | yes | 7.1 | before_cleaning | free |
| 15.10.08 | 740 | Nic | yes | 19.51 | after_cleaning | free |
| 15.10.08 | 860 | Hay | yes | 5 | before_cleaning | free |
| 16.10.08 | 480 | Wal | yes | 8.64 | before_cleaning | free |
| 16.10.08 | 805 | Gra | no | 28.62 | before_cleaning | controlled |
| 17.10.08 | 1020 | Hay | yes | 4.53 | before_cleaning | free |
| 20.10.08 | 500 | Car | yes | 18.52 | before_cleaning | controlled |
| 20.10.08 | 690 | Fer | no | 29.23 | after_training | controlled |
| 20.10.08 | 780 | Nic | yes | 10.95 | before_cleaning | free |
| 21.10.08 | 505 | Car | yes | 18.32 | before_cleaning | controlled |
| 21.10.08 | 600 | Nic | yes | 12.28 | after_cleaning | free |
| 21.10.08 | 700 | Sun | no | 15.57 | after_training | controlled |
| 21.10.08 | 720 | Fer | no | 18.41 | after_training | controlled |
| 22.10.08 | 500 | Car | yes | 22.19 | before_cleaning | controlled |
| 22.10.08 | 620 | Nic | yes | 10.18 | before_cleaning | free |
| 22.10.08 | 780 | Fer | no | 22.67 | after_training | controlled |
| 22.10.08 | 950 | Sun | no | 14.17 | after_training | controlled |

| | | | | | | |
|---|---|---|---|---|---|---|
| 23.10.08 | 500 | Car | yes | 13.99 | before_cleaning | controlled |
| 23.10.08 | 585 | Sun | no | 24.65 | after_training | controlled |
| 23.10.08 | 825 | Fer | no | 34 | after_training | controlled |
| 23.10.08 | 840 | Wal | yes | 8.45 | after_cleaning | free |
| 24.10.08 | 500 | Car | yes | 17.19 | before_cleaning | controlled |
| 24.10.08 | 910 | Hay | yes | 3.44 | before_cleaning | free |
| 24.10.08 | 930 | Fer | no | 27.78 | after_training | controlled |
| 27.10.08 | 600 | Wal | yes | 10.47 | after_cleaning | free |
| 27.10.08 | 620 | Hay | yes | 4.5 | before_cleaning | controlled |
| 27.10.08 | 625 | Tin | yes | 15.68 | after_cleaning | free |
| 28.10.08 | 520 | Fer | no | 18.57 | before_cleaning | controlled |
| 28.10.08 | 560 | Wal | yes | 8.81 | after_cleaning | controlled |
| 28.10.08 | 690 | Sun | no | 14.26 | before_cleaning | controlled |
| 28.10.08 | 840 | Nic | yes | 30.52 | before_cleaning | controlled |
| 28.10.08 | 855 | Tin | yes | 8.8 | after_cleaning | free |
| 29.10.08 | 505 | Tin | yes | 24.93 | after_cleaning | free |
| 29.10.08 | 575 | Sun | no | 17.01 | before_cleaning | controlled |
| 29.10.08 | 660 | Fer | no | 28.68 | before_cleaning | controlled |
| 29.10.08 | 800 | Wal | yes | 15.52 | after_training | controlled |
| 30.10.08 | 785 | Tin | yes | 9.49 | after_cleaning | free |
| 30.10.08 | 825 | Wal | yes | 5.38 | after_training | controlled |
| 03.11.08 | 685 | Car | yes | 11.25 | before_cleaning | controlled |
| 03.11.08 | 770 | Hay | yes | 6.67 | before_cleaning | controlled |
| 04.11.08 | 480 | Car | yes | 12.39 | before_cleaning | controlled |
| 04.11.08 | 560 | Nic | yes | 8.52 | after_cleaning | free |
| 04.11.08 | 640 | Sun | no | 6.78 | before_cleaning | controlled |
| 05.11.08 | 565 | Sun | no | 7.41 | before_cleaning | controlled |
| 05.11.08 | 710 | Car | yes | 15.66 | before_cleaning | controlled |
| 06.11.08 | 505 | Car | yes | 14.55 | before_cleaning | controlled |
| 06.11.08 | 650 | Sun | no | 16.16 | after_training | controlled |
| 06.11.08 | 840 | Syl | yes | 4.24 | before_cleaning | controlled |
| 06.11.08 | 885 | Tin | yes | 7.05 | after_cleaning | controlled |
| 07.11.08 | 500 | Car | yes | 13 | before_cleaning | controlled |
| 07.11.08 | 820 | Sun | no | 9.07 | before_cleaning | controlled |
| 07.11.08 | 900 | Hay | yes | 3.35 | after_cleaning | free |
| 10.11.08 | 765 | Syl | yes | 9.39 | before_cleaning | controlled |
| 10.11.08 | 840 | Hay | yes | 2.75 | before_cleaning | free |
| 11.11.08 | 665 | Sun | no | 6.87 | before_cleaning | controlled |
| 12.11.08 | 555 | Sun | no | 10.61 | before_cleaning | controlled |
| 12.11.08 | 585 | Fer | no | 5.6 | before_cleaning | free |
| 12.11.08 | 845 | Syl | yes | 7.88 | before_cleaning | controlled |
| 13.11.08 | 540 | Wal | yes | 6.05 | after_cleaning | free |
| 13.11.08 | 590 | Fer | no | 12.32 | before_cleaning | free |
| 14.11.08 | 620 | Nic | yes | 16.09 | after_cleaning | free |
| 14.11.08 | 750 | Sun | no | 16.92 | before_cleaning | controlled |

| | | | | | | |
|---|---|---|---|---|---|---|
| 14.11.08 | 820 | Wal | yes | 6.97 | after_cleaning | free |
| 17.11.08 | 660 | Nic | yes | 4.52 | after_cleaning | free |
| 17.11.08 | 660 | Fer | no | 4.49 | before_cleaning | controlled |
| 17.11.08 | 740 | Wal | yes | 8.6 | after_cleaning | free |
| 17.11.08 | 980 | Syl | yes | 5.65 | before_cleaning | free |
| 18.11.08 | 590 | Wal | yes | 10.93 | after_cleaning | free |
| 18.11.08 | 645 | Sun | no | 18.33 | before_cleaning | controlled |
| 18.11.08 | 720 | Nic | yes | 12.79 | after_cleaning | free |
| 18.11.08 | 940 | Syl | yes | 9.67 | before_cleaning | free |
| 19.11.08 | 600 | Sun | no | 6.37 | before_cleaning | controlled |
| 19.11.08 | 630 | Sam | no | 8.57 | after_training | controlled |
| 19.11.08 | 650 | Fer | no | 8.16 | before_cleaning | controlled |
| 19.11.08 | 830 | Car | yes | 7.23 | after_cleaning | controlled |
| 20.11.08 | 510 | Syl | yes | 23.97 | before_cleaning | free |
| 20.11.08 | 585 | Fer | no | 7.97 | before_cleaning | controlled |
| 20.11.08 | 780 | Wal | yes | 8.4 | after_cleaning | free |
| 20.11.08 | 900 | Nic | yes | 15.67 | after_cleaning | free |
| 21.11.08 | 600 | Wal | yes | 9.13 | after_cleaning | free |
| 21.11.08 | 750 | Nic | yes | 10 | after_cleaning | free |
| 24.11.08 | 520 | Hay | yes | 3.49 | after_training | controlled |
| 25.11.08 | 600 | Fer | no | 19.09 | before_cleaning | controlled |
| 26.11.08 | 570 | Syl | yes | 18.72 | after_cleaning | free |
| 27.11.08 | 590 | Syl | yes | 13.33 | after_cleaning | free |
| 27.11.08 | 820 | Car | yes | 11.7 | after_cleaning | controlled |
| 28.11.08 | 675 | Syl | yes | 13.35 | after_cleaning | free |
| 28.11.08 | 810 | Car | yes | 10.04 | after_cleaning | controlled |
| 01.12.08 | 590 | Fer | no | 9.98 | before_cleaning | controlled |
| 01.12.08 | 785 | Wal | yes | 11.31 | after_cleaning | free |
| 02.12.08 | 550 | Nic | yes | 22.54 | after_cleaning | free |
| 02.12.08 | 635 | Sun | no | 12.23 | before_cleaning | controlled |
| 02.12.08 | 725 | Tin | yes | 32.95 | before_cleaning | controlled |
| 02.12.08 | 870 | Fer | no | 7.28 | before_cleaning | controlled |
| 03.12.08 | 555 | Sun | no | 10.87 | before_cleaning | controlled |
| 04.12.08 | 540 | Sun | no | 7.24 | before_cleaning | controlled |
| 04.12.08 | 610 | Fer | no | 27.19 | before_cleaning | controlled |
| 04.12.08 | 720 | Tin | yes | 19.86 | before_cleaning | controlled |
| 05.12.08 | 510 | Sun | no | 7.29 | before_cleaning | controlled |
| 05.12.08 | 600 | Fer | no | 37.05 | before_cleaning | controlled |
| 05.12.08 | 750 | Tin | yes | 29.55 | before_cleaning | controlled |
| 08.12.08 | 810 | Fer | no | 24.84 | before_cleaning | controlled |
| 08.12.08 | 870 | Wal | yes | 9.06 | after_training | controlled |
| 09.12.08 | 645 | Sun | no | 9.96 | before_cleaning | controlled |
| 09.12.08 | 765 | Car | yes | 12.53 | before_cleaning | controlled |
| 09.12.08 | 895 | Wal | yes | 7.41 | after_cleaning | controlled |
| 10.12.08 | 540 | Sun | no | 6.73 | before_cleaning | controlled |

| | | | | | | |
|---|---|---|---|---|---|---|
| 10.12.08 | 765 | Car | yes | 10.6 | before_cleaning | controlled |
| 10.12.08 | 860 | Syl | yes | 10.22 | after_cleaning | free |
| 11.12.08 | 560 | Sun | no | 7.35 | after_training | controlled |
| 12.12.08 | 770 | Sun | no | 9.99 | before_cleaning | controlled |
| 15.12.08 | 570 | Sun | no | 6.39 | before_cleaning | controlled |
| 15.12.08 | 625 | Wal | yes | 13.21 | after_training | controlled |
| 15.12.08 | 920 | Fer | no | 15.14 | after_training | controlled |
| 16.12.08 | 660 | Sun | no | 7.91 | before_cleaning | controlled |
| 16.12.08 | 705 | Syl | yes | 14 | after_cleaning | free |
| 16.12.08 | 780 | Car | yes | 7.04 | before_cleaning | controlled |
| 16.12.08 | 1005 | Fer | no | 13.05 | after_training | controlled |
| 17.12.08 | 600 | Sun | no | 5.95 | after_training | controlled |
| 17.12.08 | 655 | Syl | yes | 14.57 | after_cleaning | free |
| 17.12.08 | 780 | Car | yes | 7.64 | before_cleaning | controlled |
| 17.12.08 | 1000 | Fer | no | 5.67 | after_training | controlled |
| 18.12.08 | 640 | Sun | no | 8.32 | after_training | controlled |
| 18.12.08 | 660 | Syl | yes | 14.44 | after_cleaning | free |
| 18.12.08 | 985 | Fer | no | 5.5 | after_training | controlled |
| 19.12.08 | 600 | Sun | no | 5.74 | after_training | controlled |
| 19.12.08 | 695 | Fer | no | 15.7 | after_training | controlled |
| 22.12.08 | 670 | Tin | yes | 10.95 | before_cleaning | free |
| 22.12.08 | 690 | Sun | no | 4.64 | before_cleaning | free |
| 22.12.08 | 750 | Pie | yes | 7.94 | after_cleaning | free |
| 22.12.08 | 765 | Syl | yes | 11.2 | after_cleaning | free |
| 23.12.08 | 690 | Tin | yes | 9.39 | after_cleaning | free |
| 24.12.08 | 550 | Car | yes | 6.52 | before_cleaning | free |
| 24.12.08 | 565 | Tin | yes | 6.59 | after_cleaning | free |
| 24.12.08 | 610 | Hay | yes | 2.37 | before_cleaning | free |
| 24.12.08 | 796 | Zor | no | 14.47 | before_cleaning | free |
| 25.12.08 | 720 | Zor | no | 6.3 | before_cleaning | free |
| 26.12.08 | 510 | Pie | yes | 10.63 | after_cleaning | free |
| 26.12.08 | 550 | Tin | yes | 4.66 | after_cleaning | free |
| 26.12.08 | 726 | Zor | no | 5.93 | before_cleaning | free |
| 27.12.08 | 690 | Car | yes | 6.01 | before_cleaning | free |
| 27.12.08 | 740 | Hay | yes | 1.79 | before_cleaning | free |
| 27.12.08 | 810 | Zor | no | 2.83 | before_cleaning | free |
| 27.12.08 | 810 | Syl | yes | 7.06 | after_cleaning | free |
| 27.12.08 | 840 | Fer | no | 3.87 | before_cleaning | free |
| 29.12.08 | 545 | Hay | yes | 6.47 | after_cleaning | free |
| 29.12.08 | 595 | Car | yes | 17.58 | after_cleaning | free |
| 29.12.08 | 600 | Fer | no | 2.92 | before_cleaning | controlled |
| 29.12.08 | 645 | Pie | yes | 11.88 | after_cleaning | free |
| 30.12.08 | 625 | Zor | no | 25.7 | before_cleaning | free |
| 30.12.08 | 640 | Tin | yes | 17.69 | before_cleaning | free |
| 30.12.08 | 750 | Pie | yes | 14.99 | after_cleaning | free |

| | | | | | | |
|---|---|---|---|---|---|---|
| 30.12.08 | 850 | Fer | no | 5.23 | before_cleaning | controlled |
| 31.12.08 | 540 | Hay | yes | 4.55 | after_cleaning | free |
| 31.12.08 | 600 | Car | yes | 13.41 | after_cleaning | free |
| 02.01.09 | 540 | Syl | yes | 5.85 | before_cleaning | free |
| 02.01.09 | 600 | Car | yes | 18.86 | after_cleaning | free |
| 02.01.09 | 670 | Pie | yes | 6.79 | after_cleaning | free |
| 02.01.09 | 690 | Fer | no | 2.17 | before_cleaning | free |
| 02.01.09 | 750 | Tin | yes | 7.76 | after_cleaning | free |
| 02.01.09 | 795 | Zor | no | 14.17 | before_cleaning | free |
| 05.01.09 | 560 | Hay | yes | 3.33 | after_cleaning | free |
| 05.01.09 | 575 | Syl | yes | 18.74 | after_cleaning | free |
| 05.01.09 | 750 | Zor | no | 12.47 | before_cleaning | free |
| 05.01.09 | 765 | Tin | yes | 4.44 | after_cleaning | free |
| 06.01.09 | 510 | Car | yes | 6.09 | after_cleaning | free |
| 06.01.09 | 520 | Tin | yes | 11.86 | after_cleaning | free |
| 06.01.09 | 545 | Nic | yes | 9.84 | after_cleaning | free |
| 06.01.09 | 600 | Sun | no | 5.2 | before_cleaning | controlled |
| 06.01.09 | 635 | Zor | no | 11.16 | before_cleaning | free |
| 06.01.09 | 635 | Syl | yes | 12.71 | after_cleaning | free |
| 06.01.09 | 720 | Fer | no | 4.59 | before_cleaning | free |
| 06.01.09 | 780 | Wal | yes | 8.45 | after_cleaning | free |
| 07.01.09 | 530 | Sun | no | 12.53 | before_cleaning | controlled |
| 07.01.09 | 540 | Syl | yes | 18.32 | after_cleaning | free |
| 07.01.09 | 780 | Nic | yes | 9.75 | after_cleaning | free |
| 07.01.09 | 825 | Fer | no | 1.89 | before_cleaning | free |
| 07.01.09 | 835 | Wal | yes | 6.87 | after_cleaning | free |
| 08.01.09 | 510 | Hay | yes | 7.69 | before_cleaning | free |
| 08.01.09 | 550 | Sun | no | 6.02 | after_training | controlled |
| 08.01.09 | 575 | Fer | no | 3.78 | before_cleaning | free |
| 08.01.09 | 585 | Nic | yes | 10.51 | after_cleaning | free |
| 08.01.09 | 615 | Wal | yes | 4.44 | before_cleaning | free |
| 08.01.09 | 645 | Wal | yes | 12.12 | after_cleaning | free |
| 08.01.09 | 645 | Pie | yes | 10.58 | after_cleaning | free |
| 08.01.09 | 670 | Syl | yes | 9.56 | after_cleaning | free |
| 08.01.09 | 743 | Zor | no | 6.85 | before_cleaning | free |
| 08.01.09 | 830 | Tin | yes | 4.41 | after_cleaning | free |
| 09.01.09 | 530 | Nic | yes | 13.16 | after_cleaning | free |
| 09.01.09 | 670 | Hay | yes | 6.95 | after_cleaning | free |
| 09.01.09 | 680 | Sun | no | 10.3 | before_cleaning | controlled |
| 09.01.09 | 720 | Pie | yes | 9.67 | after_cleaning | free |
| 09.01.09 | 720 | Syl | yes | 10.03 | after_cleaning | free |
| 09.01.09 | 795 | Tin | yes | 10.38 | after_cleaning | free |
| 09.01.09 | 840 | Car | yes | 8.81 | after_cleaning | free |
| 12.01.09 | 735 | Wal | yes | 13.08 | after_training | controlled |
| 12.01.09 | 742 | Zor | no | 8.38 | before_cleaning | free |

| | | | | | | |
|---|---|---|---|---|---|---|
| 12.01.09 | 740 | Syl | yes | 15.13 | after_cleaning | free |
| 12.01.09 | 755 | Nic | yes | 31.75 | before_cleaning | controlled |
| 12.01.09 | 795 | Hay | yes | 4.43 | before_cleaning | free |
| 12.01.09 | 805 | Fer | no | 6.98 | before_cleaning | controlled |
| 12.01.09 | 840 | Edg | no | 5.06 | before_cleaning | free |
| 12.01.09 | 860 | Luk | no | 10.72 | before_cleaning | free |
| 13.01.09 | 650 | Syl | yes | 13.9 | after_cleaning | free |
| 13.01.09 | 720 | Hay | yes | 3.83 | before_cleaning | free |
| 13.01.09 | 745 | Wal | yes | 5.47 | before_cleaning | controlled |
| 13.01.09 | 765 | Tin | yes | 27.9 | before_cleaning | controlled |
| 13.01.09 | 840 | Edg | no | 6.42 | before_cleaning | free |
| 13.01.09 | 880 | Luk | no | 11.58 | before_cleaning | free |
| 14.01.09 | 570 | Syl | yes | 10.37 | after_cleaning | free |
| 14.01.09 | 645 | Sun | no | 15.15 | after_training | controlled |
| 14.01.09 | 795 | Nic | yes | 12.87 | before_cleaning | controlled |
| 14.01.09 | 805 | Fer | no | 8.75 | before_cleaning | controlled |
| 15.01.09 | 600 | Sun | no | 9.9 | after_training | controlled |
| 15.01.09 | 590 | Fer | no | 10.13 | before_cleaning | controlled |
| 15.01.09 | 630 | Syl | yes | 14.57 | after_cleaning | free |
| 15.01.09 | 735 | Hay | yes | 12.93 | before_cleaning | free |
| 15.01.09 | 746 | Nic | yes | 10.75 | before_cleaning | controlled |
| 15.01.09 | 747 | Wal | yes | 8.03 | before_cleaning | controlled |
| 15.01.09 | 757 | Zor | no | 4.56 | before_cleaning | free |
| 16.01.09 | 575 | Syl | yes | 11.5 | after_cleaning | free |
| 16.01.09 | 690 | Sun | no | 9.17 | after_training | controlled |
| 16.01.09 | 735 | Nic | yes | 6.68 | before_cleaning | controlled |
| 16.01.09 | 735 | Wal | yes | 3.16 | before_cleaning | controlled |
| 16.01.09 | 975 | Zor | no | 3.07 | before_cleaning | free |
| 16.01.09 | 975 | Hay | yes | 3.34 | before_cleaning | free |
| 17.01.09 | 540 | Fer | no | 9.4 | before_cleaning | controlled |
| 18.01.09 | 540 | Fer | no | 7.96 | before_cleaning | controlled |
| 19.01.09 | 685 | Sun | no | 18.08 | after_training | controlled |
| 19.01.09 | 695 | Syl | yes | 22.54 | after_cleaning | free |
| 19.01.09 | 725 | Wal | yes | 11.56 | after_training | controlled |
| 19.01.09 | 840 | Hay | yes | 5.51 | before_cleaning | free |
| 20.01.09 | 560 | Fer | no | 15.99 | after_training | controlled |
| 20.01.09 | 720 | Sun | no | 5.95 | after_training | controlled |
| 20.01.09 | 860 | Zor | no | 2.83 | before_cleaning | free |
| 21.01.09 | 615 | Sun | no | 6.63 | after_training | controlled |
| 21.01.09 | 820 | Hay | yes | 4.33 | before_cleaning | free |
| 22.01.09 | 650 | Syl | yes | 10.61 | after_cleaning | free |
| 22.01.09 | 690 | Sun | no | 9.06 | after_training | controlled |
| 22.01.09 | 795 | Fer | no | 27.58 | before_cleaning | controlled |
| 22.01.09 | 585 | Zor | no | 11.62 | before_cleaning | free |
| 23.01.09 | 590 | Fer | no | 19.66 | before_cleaning | controlled |

| | | | | | | |
|---|---|---|---|---|---|---|
| 23.01.09 | 615 | Syl | yes | 10.68 | after_cleaning | free |
| 23.01.09 | 780 | Sun | no | 6.99 | before_cleaning | controlled |
| 23.01.09 | 820 | Hay | yes | 2.61 | before_cleaning | free |
| 25.01.09 | 790 | Zor | no | 3.8 | before_cleaning | free |
| 26.01.09 | 700 | Car | yes | 15.19 | after_cleaning | free |
| 26.01.09 | 715 | Syl | yes | 14.81 | after_cleaning | free |
| 26.01.09 | 720 | Pie | yes | 12.2 | after_cleaning | free |
| 26.01.09 | 740 | Wal | yes | 4.65 | before_cleaning | controlled |
| 26.01.09 | 755 | Nic | yes | 12.72 | before_cleaning | free |
| 26.01.09 | 770 | Zor | no | 5.89 | before_cleaning | free |
| 26.01.09 | 810 | Sun | no | 5.97 | before_cleaning | free |
| 26.01.09 | 870 | Hay | yes | 4.49 | before_cleaning | free |
| 27.01.09 | 600 | Syl | yes | 15.13 | after_cleaning | free |
| 27.01.09 | 665 | Sun | no | 5.4 | before_cleaning | free |
| 27.01.09 | 725 | Car | yes | 11.5 | before_cleaning | controlled |
| 27.01.09 | 731 | Wal | yes | 4.55 | before_cleaning | controlled |
| 27.01.09 | 749 | Nic | yes | 6.85 | before_cleaning | free |
| 28.01.09 | 580 | Fer | no | 12.03 | before_cleaning | controlled |
| 28.01.09 | 630 | Pie | yes | 12.34 | after_cleaning | free |
| 28.01.09 | 730 | Zor | no | 5.6 | before_cleaning | free |
| 29.01.09 | 810 | Car | yes | 12.76 | before_cleaning | controlled |
| 30.01.09 | 630 | Pie | yes | 19.64 | after_cleaning | free |
| 30.01.09 | 720 | Syl | yes | 13.17 | after_cleaning | free |
| 30.01.09 | 795 | Car | yes | 7.46 | before_cleaning | controlled |
| 31.01.09 | 460 | Fer | no | 13.49 | before_cleaning | controlled |
| 01.02.09 | 580 | Fer | no | 9.15 | before_cleaning | controlled |
| 02.02.09 | 645 | Syl | yes | 18.88 | after_cleaning | free |
| 02.02.09 | 710 | Hay | yes | 8.64 | after_cleaning | controlled |
| 02.02.09 | 710 | Sun | no | 10.61 | after_training | controlled |
| 02.02.09 | 716 | Wal | yes | 12.84 | before_cleaning | controlled |
| 02.02.09 | 840 | Edg | no | 9.42 | before_cleaning | free |
| 02.02.09 | 760 | Tin | yes | 14.15 | after_cleaning | controlled |
| 02.02.09 | 760 | Nic | yes | 19.63 | after_cleaning | free |
| 03.02.09 | 680 | Sun | no | 8.3 | after_training | controlled |
| 03.02.09 | 690 | Zor | no | 7.9 | before_cleaning | free |
| 03.02.09 | 800 | Car | yes | 8.65 | before_cleaning | controlled |
| 03.02.09 | 820 | Wal | yes | 6.2 | before_cleaning | controlled |
| 03.02.09 | 840 | Edg | no | 13.88 | before_cleaning | free |
| 03.02.09 | 850 | Luk | no | 10.64 | before_cleaning | free |
| 04.02.09 | 700 | Sun | no | 7.21 | after_training | controlled |
| 04.02.09 | 770 | Tin | yes | 12.11 | before_cleaning | controlled |
| 04.02.09 | 800 | Car | yes | 7.66 | before_cleaning | controlled |
| 04.02.09 | 815 | Fer | no | 4.57 | before_cleaning | controlled |
| 04.02.09 | 820 | Syl | yes | 9.48 | after_cleaning | controlled |
| 04.02.09 | 870 | Edg | no | 9.16 | before_cleaning | free |

| | | | | | | |
|---|---|---|---|---|---|---|
| 04.02.09 | 880 | Luk | no | 8.49 | before_cleaning | free |
| 05.02.09 | 645 | Fer | no | 8.46 | before_cleaning | controlled |
| 05.02.09 | 780 | Tin | yes | 19.46 | before_cleaning | controlled |
| 05.02.09 | 805 | Car | yes | 8.56 | before_cleaning | controlled |
| 05.02.09 | 819 | Syl | yes | 12.47 | after_cleaning | controlled |
| 05.02.09 | 675 | Sun | no | 12.4 | after_training | controlled |
| 06.02.09 | 810 | Edg | no | 11.27 | before_cleaning | free |
| 06.02.09 | 820 | Car | yes | 9.36 | before_cleaning | controlled |
| 06.02.09 | 860 | Pie | yes | 24.44 | after_cleaning | free |
| 09.02.09 | 550 | Hay | yes | 10.37 | after_cleaning | free |
| 09.02.09 | 640 | Fer | no | 3.52 | before_cleaning | controlled |
| 09.02.09 | 705 | Sun | no | 5.13 | after_training | controlled |
| 09.02.09 | 745 | Sam | no | 13.88 | before_cleaning | controlled |
| 09.02.09 | 795 | Luk | no | 9.26 | before_cleaning | free |
| 09.02.09 | 800 | Edg | no | 14.59 | before_cleaning | free |
| 10.02.09 | 625 | Zor | no | 11.28 | before_cleaning | free |
| 10.02.09 | 750 | Sun | no | 12.75 | after_training | controlled |
| 10.02.09 | 820 | Sam | no | 9.52 | after_training | controlled |
| 10.02.09 | 825 | Car | yes | 9.05 | before_cleaning | controlled |
| 11.02.09 | 705 | Sun | no | 12.57 | after_training | controlled |
| 11.02.09 | 810 | Tin | yes | 19.79 | before_cleaning | controlled |
| 11.02.09 | 840 | Luk | no | 6.52 | before_cleaning | free |
| 12.02.09 | 685 | Car | yes | 8.08 | before_cleaning | controlled |
| 12.02.09 | 825 | Fer | no | 20.52 | before_cleaning | controlled |
| 12.02.09 | 890 | Sam | no | 37.62 | before_cleaning | controlled |
| 13.02.09 | 735 | Wal | yes | 5.71 | before_cleaning | controlled |
| 13.02.09 | 735 | Tin | yes | 29.97 | before_cleaning | controlled |
| 13.02.09 | 740 | Nic | yes | 23.01 | before_cleaning | controlled |
| 13.02.09 | 780 | Sun | no | 10.72 | after_training | controlled |
| 13.02.09 | 810 | Car | yes | 11.62 | before_cleaning | controlled |
| 13.02.09 | 830 | Fer | no | 29.83 | before_cleaning | controlled |
| 13.02.09 | 850 | Edg | no | 9.43 | before_cleaning | free |
| 13.02.09 | 870 | Luk | no | 15 | before_cleaning | free |
| 14.02.09 | 450 | Fer | no | 13.44 | before_cleaning | controlled |
| 16.02.09 | 675 | Sun | no | 7.85 | after_training | controlled |
| 16.02.09 | 720 | Fer | no | 30.05 | before_cleaning | controlled |
| 16.02.09 | 735 | Tin | yes | 23.5 | before_cleaning | controlled |
| 16.02.09 | 785 | Nic | yes | 17.13 | before_cleaning | controlled |
| 16.02.09 | 825 | Sam | no | 11.18 | before_cleaning | controlled |
| 16.02.09 | 830 | Luk | no | 18.18 | before_cleaning | free |
| 17.02.09 | 670 | Luk | no | 2.9 | before_cleaning | free |
| 17.02.09 | 805 | Sam | no | 15.2 | before_cleaning | controlled |
| 17.02.09 | 870 | Sun | no | 13.15 | after_training | controlled |
| 18.02.09 | 645 | Edg | no | 9.39 | before_cleaning | free |
| 18.02.09 | 675 | Fer | no | 11.16 | before_cleaning | controlled |

| | | | | | | |
|---|---|---|---|---|---|---|
| 18.02.09 | 690 | Sun | no | 22.21 | after_training | controlled |
| 18.02.09 | 750 | Tin | yes | 28.97 | before_cleaning | controlled |
| 18.02.09 | 830 | Luk | no | 10.33 | before_cleaning | free |
| 18.02.09 | 830 | Car | yes | 7.07 | before_cleaning | controlled |
| 19.02.09 | 530 | Fer | no | 12.29 | before_cleaning | controlled |
| 19.02.09 | 715 | Sun | no | 8.31 | after_training | controlled |
| 19.02.09 | 735 | Tin | yes | 39.2 | before_cleaning | controlled |
| 20.02.09 | 590 | Fer | no | 7.65 | before_cleaning | controlled |
| 20.02.09 | 750 | Tin | yes | 29.27 | before_cleaning | controlled |
| 20.02.09 | 795 | Car | yes | 10.06 | before_cleaning | controlled |
| 23.02.09 | 540 | Hay | yes | 9.12 | after_cleaning | free |
| 23.02.09 | 770 | Tin | yes | 16.4 | before_cleaning | controlled |
| 24.02.09 | 750 | Tin | yes | 21.7 | before_cleaning | controlled |
| 24.02.09 | 795 | Car | yes | 6.39 | before_cleaning | controlled |
| 24.02.09 | 820 | Fer | no | 7.89 | before_cleaning | controlled |
| 25.02.09 | 540 | Fer | no | 6.77 | before_cleaning | controlled |
| 25.02.09 | 710 | Syl | yes | 19.73 | after_cleaning | controlled |
| 25.02.09 | 760 | Tin | yes | 29.86 | before_cleaning | controlled |
| 25.02.09 | 875 | Sam | no | 5.55 | before_cleaning | controlled |
| 26.02.09 | 690 | Syl | yes | 17.52 | after_cleaning | controlled |
| 26.02.09 | 720 | Tin | yes | 27.39 | before_cleaning | controlled |
| 26.02.09 | 830 | Car | yes | 6.91 | before_cleaning | controlled |
| 26.02.09 | 840 | Sam | no | 8.51 | before_cleaning | controlled |
| 27.02.09 | 675 | Wal | yes | 7.75 | before_cleaning | controlled |
| 27.02.09 | 715 | Sam | no | 9.31 | before_cleaning | controlled |
| 27.02.09 | 770 | Tin | yes | 30.19 | before_cleaning | controlled |
| 27.02.09 | 820 | Car | yes | 6.35 | before_cleaning | controlled |
| 01.03.09 | 700 | Fer | no | 3.34 | before_cleaning | free |
| 02.03.09 | 550 | Fer | no | 3.23 | before_cleaning | controlled |
| 02.03.09 | 680 | Wal | yes | 7.69 | after_training | controlled |
| 02.03.09 | 760 | Tin | yes | 9.33 | before_cleaning | controlled |
| 02.03.09 | 765 | Car | yes | 10.15 | before_cleaning | controlled |
| 02.03.09 | 825 | Sun | no | 6.72 | after_training | controlled |
| 02.03.09 | 840 | Luk | no | 11.46 | before_cleaning | free |
| 02.03.09 | 855 | Edg | no | 4.44 | before_cleaning | free |
| 03.03.09 | 738 | Sam | no | 28.59 | before_cleaning | controlled |
| 03.03.09 | 750 | Car | yes | 7.24 | before_cleaning | controlled |
| 03.03.09 | 795 | Sun | no | 9.92 | after_training | controlled |
| 03.03.09 | 795 | Tin | yes | 10.22 | before_cleaning | controlled |
| 03.03.09 | 850 | Fer | no | 8.9 | before_cleaning | controlled |
| 04.03.09 | 750 | Sam | no | 7.5 | before_cleaning | controlled |
| 04.03.09 | 795 | Tin | yes | 10.81 | before_cleaning | controlled |
| 04.03.09 | 805 | Fer | no | 3.77 | before_cleaning | controlled |
| 04.03.09 | 930 | Sun | no | 7.03 | after_training | controlled |
| 05.03.09 | 790 | Sun | no | 6.97 | after_training | controlled |

| | | | | | | |
|---|---|---|---|---|---|---|
| 05.03.09 | 810 | Sam | no | 7.5 | before_cleaning | controlled |
| 06.03.09 | 565 | Sam | no | 20.68 | before_cleaning | controlled |
| 06.03.09 | 740 | Car | yes | 17.3 | before_cleaning | controlled |
| 06.03.09 | 810 | Sun | no | 8.54 | after_training | controlled |
| 06.03.09 | 850 | Fer | no | 12.13 | before_cleaning | controlled |
| 08.03.09 | 840 | Fer | no | 2.27 | before_cleaning | free |
| 09.03.09 | 720 | Sun | no | 6.15 | after_training | controlled |
| 09.03.09 | 725 | Fer | no | 2.01 | before_cleaning | free |
| 09.03.09 | 785 | Wal | yes | 4.87 | before_cleaning | controlled |
| 09.03.09 | 820 | Luk | no | 5.34 | before_cleaning | free |
| 09.03.09 | 840 | Edg | no | 5.36 | before_cleaning | free |
| 10.03.09 | 530 | Sam | no | 9.13 | before_cleaning | controlled |
| 10.03.09 | 775 | Car | yes | 10.28 | before_cleaning | controlled |
| 10.03.09 | 815 | Edg | no | 4.18 | before_cleaning | free |
| 10.03.09 | 810 | Sun | no | 6.6 | after_training | controlled |
| 11.03.09 | 600 | Fer | no | 15.79 | before_cleaning | free |
| 11.03.09 | 730 | Sun | no | 6.62 | after_training | controlled |
| 11.03.09 | 780 | Edg | no | 4.75 | before_cleaning | free |
| 11.03.09 | 805 | Wal | yes | 5.61 | before_cleaning | controlled |
| 12.03.09 | 600 | Fer | no | 3.04 | before_cleaning | free |
| 12.03.09 | 695 | Car | yes | 10.74 | before_cleaning | controlled |
| 12.03.09 | 720 | Sun | no | 7.59 | after_training | controlled |
| 12.03.09 | 860 | Edg | no | 4.65 | before_cleaning | free |
| 13.03.09 | 530 | Fer | no | 3.1 | before_cleaning | free |
| 13.03.09 | 545 | Sam | no | 12 | before_cleaning | controlled |
| 13.03.09 | 705 | Car | yes | 7.97 | before_cleaning | controlled |
| 14.03.09 | 485 | Fer | no | 2.54 | before_cleaning | free |
| 15.03.09 | 590 | Fer | no | 4.25 | before_cleaning | free |
| 16.03.09 | 680 | Wal | yes | 7.85 | after_cleaning | free |
| 16.03.09 | 755 | Nic | yes | 20.63 | after_cleaning | free |
| 16.03.09 | 860 | Fer | no | 9.75 | after_training | controlled |
| 16.03.09 | 870 | Edg | no | 5.85 | before_cleaning | free |
| 16.03.09 | 900 | Luk | no | 12.47 | before_cleaning | free |
| 16.03.09 | 910 | Sun | no | 4.02 | after_training | controlled |
| 17.03.09 | 520 | Sam | no | 20.1 | before_cleaning | free |
| 17.03.09 | 750 | Sun | no | 6 | after_training | controlled |
| 17.03.09 | 800 | Edg | no | 5.41 | before_cleaning | free |
| 17.03.09 | 815 | Luk | no | 6.04 | before_cleaning | free |
| 18.03.09 | 535 | Fer | no | 12.29 | before_cleaning | controlled |
| 18.03.09 | 665 | Wal | yes | 8.21 | after_training | controlled |
| 18.03.09 | 700 | Hay | yes | 4.26 | after_cleaning | controlled |
| 18.03.09 | 780 | Luk | no | 5.72 | before_cleaning | free |
| 18.03.09 | 820 | Edg | no | 11.37 | before_cleaning | free |
| 18.03.09 | 870 | Sun | no | 6.39 | after_training | controlled |
| 19.03.09 | 780 | Sun | no | 5.18 | after_training | controlled |

| | | | | | | |
|---|---|---|---|---|---|---|
| 20.03.09 | 840 | Sun | no | 4.99 | after_training | controlled |
| 23.03.09 | 420 | Hay | yes | 9.53 | after_cleaning | free |
| 23.03.09 | 465 | Car | yes | 12.08 | after_cleaning | free |
| 23.03.09 | 520 | Fer | no | 3.08 | before_cleaning | free |
| 23.03.09 | 645 | Nic | yes | 12.25 | after_cleaning | free |
| 25.03.09 | 525 | Fer | no | 6.38 | before_cleaning | free |
| 27.03.09 | 740 | Fer | no | 11.69 | before_cleaning | free |
| 30.03.09 | 745 | Hay | yes | 10.77 | after_cleaning | controlled |
| 30.03.09 | 805 | Car | yes | 23.49 | after_cleaning | free |
| 30.03.09 | 790 | Edg | no | 11.07 | before_cleaning | free |
| 30.03.09 | 840 | Sun | no | 12.6 | after_training | controlled |
| 30.03.09 | 860 | Sam | no | 28.09 | before_cleaning | free |
| 31.03.09 | 830 | Car | yes | 5.61 | before_cleaning | free |
| 31.03.09 | 930 | Sun | no | 5.71 | after_training | controlled |
| 01.04.09 | 540 | Fer | no | 9.45 | before_cleaning | free |
| 01.04.09 | 810 | Sun | no | 10.23 | after_training | controlled |
| 01.04.09 | 835 | Hay | yes | 5.11 | before_cleaning | free |
| 02.04.09 | 725 | Sam | no | 12.51 | before_cleaning | controlled |
| 02.04.09 | 735 | Nic | yes | 13.29 | before_cleaning | controlled |
| 02.04.09 | 820 | Sun | no | 9.87 | after_training | controlled |
| 03.04.09 | 660 | Sam | no | 9.24 | before_cleaning | controlled |
| 03.04.09 | 690 | Car | yes | 8.73 | before_cleaning | free |
| 03.04.09 | 850 | Hay | yes | 4.68 | before_cleaning | free |
| 03.04.09 | 1050 | Sun | no | 7.41 | after_training | controlled |
| 06.04.09 | 720 | Sam | no | 11.91 | before_cleaning | controlled |
| 06.04.09 | 735 | Sun | no | 16.92 | after_training | controlled |
| 06.04.09 | 740 | Hay | yes | 12.51 | before_cleaning | free |
| 06.04.09 | 780 | Pie | yes | 16.15 | after_cleaning | controlled |
| 06.04.09 | 900 | Car | yes | 7.09 | before_cleaning | free |
| 07.04.09 | 540 | Fer | no | 5.2 | before_cleaning | free |
| 07.04.09 | 577 | Sam | no | 17.47 | before_cleaning | controlled |
| 07.04.09 | 960 | Sun | no | 4.22 | after_training | controlled |
| 08.04.09 | 740 | Hay | yes | 13.35 | after_cleaning | free |
| 08.04.09 | 785 | Car | yes | 5.6 | before_cleaning | free |
| 08.04.09 | 900 | Sun | no | 5.82 | after_training | controlled |
| 08.04.09 | 915 | Zor | no | 12.36 | before_cleaning | free |
| 10.04.09 | 705 | Hay | yes | 12.94 | before_cleaning | free |
| 11.04.09 | 820 | Fer | no | 1.36 | before_cleaning | free |
| 12.04.09 | 760 | Hay | yes | 6.64 | after_cleaning | free |
| 12.04.09 | 785 | Car | yes | 17.66 | after_cleaning | free |
| 12.04.09 | 785 | Fer | no | 2.4 | before_cleaning | free |
| 13.04.09 | 800 | Fer | no | 2.36 | before_cleaning | free |
| 14.04.09 | 550 | Fer | no | 6.64 | before_cleaning | controlled |
| 14.04.09 | 755 | Sam | no | 19.06 | before_cleaning | controlled |
| 14.04.09 | 885 | Sun | no | 8.99 | after_training | controlled |

| | | | | | | |
|---|---|---|---|---|---|---|
| 14.04.09 | 900 | Car | yes | 5.45 | before_cleaning | free |
| 14.04.09 | 1080 | Hay | yes | 7.14 | before_cleaning | free |
| 15.04.09 | 510 | Sam | no | 23.1 | before_cleaning | controlled |
| 15.04.09 | 600 | Car | yes | 12.16 | after_cleaning | free |
| 15.04.09 | 790 | Tin | yes | 13.36 | before_cleaning | controlled |
| 15.04.09 | 815 | Nic | yes | 20.28 | after_cleaning | controlled |
| 15.04.09 | 855 | Sun | no | 3.68 | after_training | controlled |
| 15.04.09 | 920 | Zor | no | 6.41 | before_cleaning | free |
| 16.04.09 | 505 | Fer | no | 17.58 | before_cleaning | controlled |
| 16.04.09 | 741 | Sam | no | 21.74 | before_cleaning | controlled |
| 16.04.09 | 840 | Sun | no | 4.05 | before_cleaning | controlled |
| 17.04.09 | 515 | Fer | no | 31.82 | before_cleaning | controlled |
| 17.04.09 | 660 | Sun | no | 5.15 | after_training | controlled |
| 17.04.09 | 765 | Car | yes | 18.48 | after_cleaning | free |
| 17.04.09 | 790 | Hay | yes | 2.67 | before_cleaning | free |
| 20.04.09 | 495 | Fer | no | 6.86 | before_cleaning | controlled |
| 20.04.09 | 615 | Car | yes | 20.62 | after_cleaning | free |
| 20.04.09 | 715 | Sun | no | 11.76 | after_training | controlled |
| 20.04.09 | 810 | Hay | yes | 5.52 | before_cleaning | free |
| 20.04.09 | 830 | Tin | yes | 10.21 | after_cleaning | free |
| 20.04.09 | 923 | Zor | no | 15.26 | before_cleaning | free |
| 21.04.09 | 525 | Fer | no | 28.58 | before_cleaning | controlled |
| 21.04.09 | 670 | Sun | no | 7.84 | after_training | controlled |
| 21.04.09 | 800 | Sam | no | 10.29 | before_cleaning | controlled |
| 21.04.09 | 833 | Zor | no | 8.24 | before_cleaning | free |
