# Supplementary material for: Assessment of stress responses in rhesus macaques (Macaca mulatta) to daily routine procedures in system neuroscience based on salivary cortisol concentrations
Source: PLoS One. 2018 Jan 2;13(1):e0190190. doi: 10.1371/journal.pone.0190190 (PMC5749769; doi:10.1371/journal.pone.0190190)
Supplement: S1 Table — Mean cortisol values (ln-transformed) per animal and condition. (PDF) [file pone.0190190.s002.pdf]

S1 Table. Summary of cortisol values per animal and condition. Mean cortisol values (ln-transformed) per animal and condition.

	before cleaning		after cleaning		after training
**individual**	**free fluid**	**contr. fluid**	**free fluid**	**contr. fluid**	**contr. fluid**
Car	1.97	2.38	2.67	2.40	
Edg	2.00				
Fer	1.36	2.46			2.67
Gra		2.91			3.05
Hay	1.70	1.70	1.89	1.99	1.25
Luk	2.32				
Mar		2.31			
Nic	2.38	2.78	2.54	3.01	
Pie	1.21	2.86	2.46	2.77	
Sam	3.17	2.58			2.20
Sun	1.67	2.42			2.28
Syl	2.20	2.65	2.59	2.65	
Tin	2.25	2.97	2.32	2.40	
Wal	2.05	1.86	2.16	1.95	2.06
Zor	2.04				
